# Role of pleiotropy during adaptation of TEM-1 *β*-lactamase to two novel antibiotics

**DOI:** 10.1111/eva.12200

**Published:** 2014-09-18

**Authors:** Martijn F Schenk, Sariette Witte, Merijn L M Salverda, Bertha Koopmanschap, Joachim Krug, J Arjan G M de Visser

**Affiliations:** 1 Institute of Genetics, University of CologneKöln, Germany; 2 Laboratory of Genetics, Wageningen UniversityWageningen, The Netherlands; 3 Institute for Theoretical Physics, University of CologneKöln, Germany; 4 Systems Biology of Ageing Cologne (Sybacol), University of CologneKöln, Germany

**Keywords:** antibiotic resistance, epistasis, pleiotropy, protein evolution, TEM-1 *β*-lactamase, trade-off

## Abstract

Pleiotropy is a key feature of the genotype–phenotype map, and its form and extent have many evolutionary implications, including for the dynamics of adaptation and the evolution of specialization. Similarly, pleiotropic effects of antibiotic resistance mutations may affect the evolution of antibiotic resistance in the simultaneous or fluctuating presence of different antibiotics. Here, we study the role of pleiotropy during the *in vitro* adaptation of the enzyme TEM-1 *β*-lactamase to two novel antibiotics, cefotaxime (CTX) and ceftazidime (CAZ). We subject replicate lines for four rounds of evolution to selection with CTX and CAZ alone, and in their combined and fluctuating presence. Evolved alleles show positive correlated responses when selecting with single antibiotics. Nevertheless, pleiotropic constraints are apparent from the effects of single mutations and from selected alleles showing smaller correlated than direct responses and smaller responses after simultaneous and fluctuating selection with both than with single antibiotics. We speculate that these constraints result from structural changes in the oxyanion pocket surrounding the active site, where accommodation of CTX and the larger CAZ is balanced against their positioning with respect to the active site. Our findings suggest limited benefits from the combined or fluctuating application of these related cephalosporins for containing antibiotic resistance.

## Introduction

Understanding the causal connections between genotype and phenotype is one of the grand topics in biology. Key questions about the mapping between genotype and phenotype are how multiple loci interact to produce a phenotype (epistasis) and how single loci affect multiple phenotypes (pleiotropy). Epistasis and pleiotropy are important sources of evolutionary constraint, which affect the course and outcome of evolution (Lenski 1988; Otto 2004; Østman et al. 2011; de Visser et al. 2011; Remold 2012; de Visser and Krug 2014). Moreover, epistasis and pleiotropy are interconnected features, because both arise from the molecular interactions that underlie biological functions and are affected by evolutionary historical contingencies and physicochemical constraints (Maynard Smith et al. 1985; Novak et al. 2006).

The evolutionary consequences of pleiotropy are particularly apparent when the different phenotypic effects have opposite effects on the fitness of the organism, called antagonistic (Williams 1957) or sign pleiotropy – in analogy to terminology used for categorizing epistasis (Remold 2012). Sign pleiotropy results from trade-offs between phenotypic traits and may explain the evolution of aging (Williams 1957), different life histories (Stearns 1992), and niche or resource specialization (Futuyma and Moreno 1988; Kassen 2002; Remold 2012). However, pleiotropy can also be of the magnitude type (Remold 2012), where both direct and correlated responses are positive, but the latter is often smaller in effect than the former. For example, beneficial mutations in *Escherichia coli* selected in a glucose-limited environment were also beneficial in the presence of other sugars, although mostly of smaller benefit (Ostrowski et al. 2005; Leiby and Marx 2014). Pleiotropy also plays a central role in Fisher's geometric model of adaptation, which explains limitations on the rate of adaptation due to pleiotropic constraints on effect sizes of beneficial mutations (Fisher 1930).

At a fundamental level, trade-offs causing antagonistic pleiotropy result from structural, developmental, or thermodynamic constraints. As an example, the difference in free energy of substrate and product in an ATP-producing pathway limits the allocation of energy into ATP production versus driving the pathway, which may explain the trade-off between microbial growth rate and yield (Pfeiffer et al. 2001). Other examples are energy or metabolic budgets explaining the allocation of resources to different life-history traits (van Noordwijk and de Jong 1986), such as to female or male reproduction in the hermaphroditic pond snail *Lymnea stagnalis* (de Visser et al. 1994). Trade-offs exist even at the level of single proteins, such as between protein activity and thermodynamic stability mediated by structural constraints (Wang et al. 2002; DePristo et al. 2005).

Pleiotropy is also a key determinant in the evolution of antibiotic resistance. Pleiotropic effects of resistance mutations are apparent from the so-called cost of resistance in the absence of antibiotics due to the allocation of resources into antibiotic-degrading enzymes or efflux pumps, or compromised cellular functions, such as DNA or protein synthesis (Andersson and Hughes 2010). However, pleiotropy may also constrain selection in the simultaneous or fluctuating presence of different antibiotics, or – in contrast – facilitate escape from epistatic constraints to adaptation in the presence of a single antibiotic (Goulart et al. 2013; Schenk and De Visser 2013). Consequently, knowledge about pleiotropic constraints might help to contain the problem of resistance by designing smart drug therapies, for instance using drug combinations where resistance to one drug increases or maintains sensitivity to another drug (MacLean et al. 2010; Goulart et al. 2013; Imamovic and Sommer 2013; Palmer and Kishony 2013; Pena-Miller et al. 2013; Schenk and De Visser 2013; Jansen et al. 2014).

**Figure 1:**
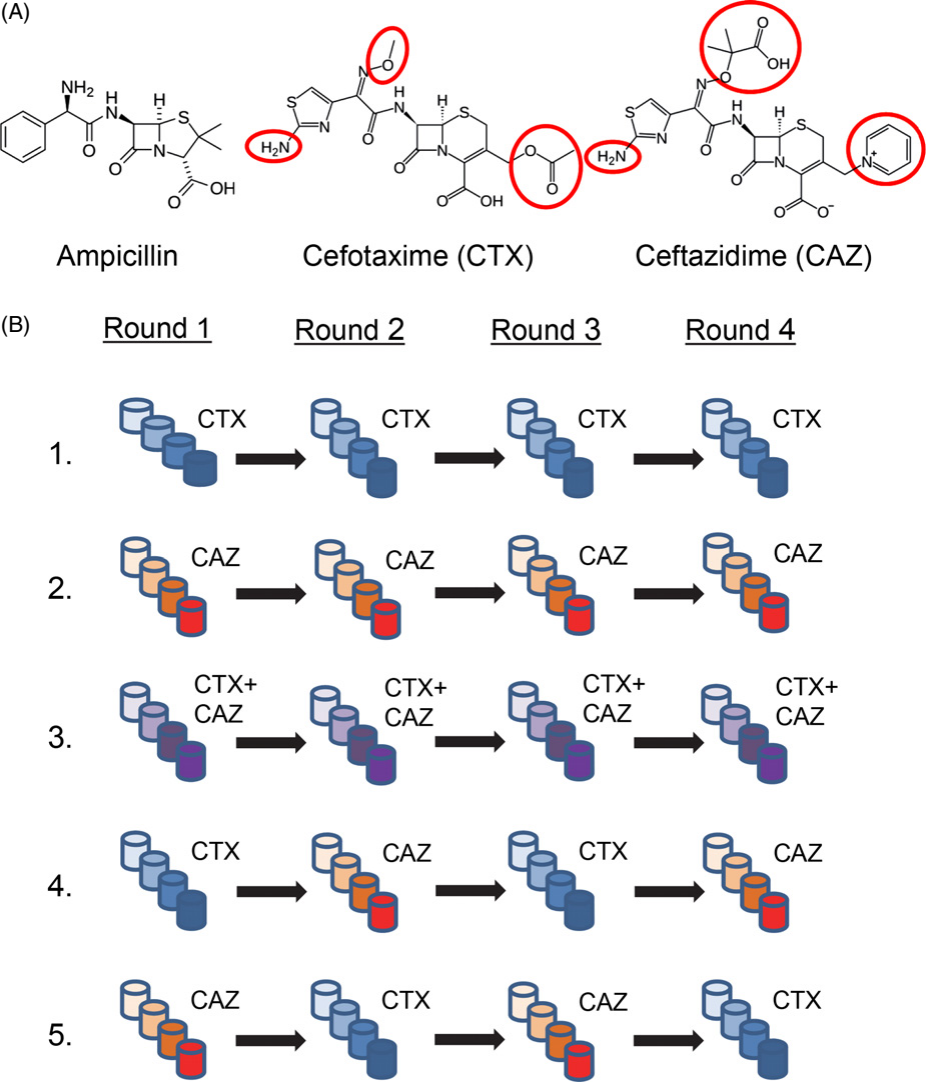
Experimental system and setup. (A) Structure of the antibiotics ampicillin, cefotaxime and ceftazidime. The enzyme TEM-1 that we used confers high resistance to ampicillin and low resistance toward CTX and CAZ. These antibiotics are larger than ampicillin and have side chains (encircled in red) that may interfere with the positioning of the antibiotic into the oxyanion pocket that surrounds the catalytic site (Vakulenko et al. 1999; Orencia et al. 2001). The side chains are longer in CAZ, which may give rise to pleiotropy. (B) Experimental evolution conditions. Five conditions were tested in which six replicate experimental lines were exposed to four rounds of error-prone PCR followed by selection with increasing concentrations (shown by increased darkness of selection cultures) of the *β*-lactam antibiotics CTX (blue), CAZ (red), or a combination of both (purple).

Here, we study the role of pleiotropy during the evolution of resistance conferred by TEM-1 *β*-lactamase to two novel antibiotics. TEM-1 has played an important role in the emergence of extended-spectrum *β*-lactamases (Salverda et al. 2010). It has high activity on ampicillin and promiscuous (i.e., low, but nonzero) activity on ‘extended-spectrum’ cephalosporins cefotaxime (CTX) and ceftazidime (CAZ), but can substantially increase activity on these novel antibiotics through the substitution of a wide range of mutations (Blazquez et al. 2000; Fujii et al. 2004; Salverda et al. 2011; Schenk et al. 2012). We expect pleiotropic constraints of mutations affecting resistance to these antibiotics, based on their different sizes and chemical structures (see Fig.1A) and the observation that different mutations are selected in the presence of these antibiotics: preferably at amino acid position 238 for CTX (Salverda et al. 2011) and at position 164 for CAZ (Blazquez et al. 2000; Fujii et al. 2004). However, we do not know the extent of these pleiotropic constraints; that is, whether pleiotropy is of the sign or magnitude type.

We test for the role of pleiotropy during adaptation of TEM-1 to CTX and CAZ under various conditions guided by three questions. First, we ask whether mutations that are selected in the presence of a single antibiotic show pleiotropic effects that reflect a trade-off between adaptation to CTX and CAZ. Second, we ask whether and how pleiotropic effects of mutations selected in the presence of both CTX and CAZ affect adaptation to these antibiotics. Finally, we ask whether selection in the fluctuating presence of CTX and CAZ constrains adaptation or rather releases it from epistatic constraints experienced in the presence of a single antibiotic, as observed for adaptation to CTX (Weinreich et al. 2006; Salverda et al. 2011; Schenk et al. 2013). Our results reveal negative magnitude pleiotropy of selected alleles, as well as sign pleiotropy of individual mutations. Magnitude pleiotropy is asymmetric, because selection for CTX resistance is accompanied by smaller increases in CAZ resistance than vice versa. We discuss likely causes for these results, as well as general implications for the containment of antibiotic resistance.

## Materials and methods

*Escherichia coli* strain DH5*α*E (Invitrogen, Carlsbad, CA, USA) was used as the host for all plasmids. The pACSE3 plasmid was used as a vector for cloning and expression of TEM *β*-lactamase alleles (Bolivar et al. 1977; Barlow and Hall 2002). pACSE3 is present at ∼10 copies per cell and carries a tetracycline resistance gene and *lacI*. TEM-1 was amplified from pBR322 and cloned into the pACSE3 plasmid to yield pACTEM1 (Barlow and Hall 2002).

### Experimental evolution conditions

We studied the evolution of plasmid-borne TEM-1 *β*-lactamase in response to two *β*-lactam antibiotics across four rounds of error-prone PCR mutagenesis followed by selection after expression in bacterial hosts. Mutations were randomly introduced by error-prone PCR, and mutants with increased antibiotic resistance were selected using cefotaxime (CTX; Sigma, St. Louis, MO, USA) and ceftazidime (CAZ; Sigma). Experimental lines were exposed to five experimental conditions for four rounds of selection (Fig.1). For each experimental condition, we used six replicate lines. Mutants with increased resistance were selected with CTX or CAZ alone, with both antibiotics simultaneously or by alternating between CTX and CAZ, while using either CTX or CAZ in the initial selection round. Because the initial MICs of CTX (0.125 *μ*g/mL) and CAZ (1 *μ*g/mL) differed by a factor eight, we used a 1:8 ratio between these antibiotics when combining CTX and CAZ.

### Mutagenesis

We introduced random mutations into TEM-1 using the GeneMorph II random mutagenesis kit (Stratagene). The primers P3 (TCATCCGGCTCGTATAATGTGTGGA) and P4 (ACTCTCTTCCGGGCGCTATCAT) flank the multiple cloning site of pACSE3 and were used for PCR amplification with pACTEM1 as a template. The mutagenesis conditions were tuned to induce ∼2 mutations per amplicon by adding 1130 ng of plasmid template (Salverda et al. 2011). The cycling program consisted of the following: denaturation at 95°C for 2 min, 30 cycles of denaturation (30 s at 95°C), annealing (30 s at 60°C), and extension (75 s at 72°C), followed by a final step at 72°C for 10 min.

Amplicons were digested with BspHI and SacI restriction enzymes, followed by DpnI (to remove template), ligated into pACSE3 using T4 DNA ligase (New England Biolabs, Ipswich, MA, USA), and electroporated into DH5*α*E. Cells were allowed to recover in SOC medium (20 g tryptone and 5 g yeast extract/liter supplied with 10 mm NaCl, 2.5 mm KCl, 10 mm MgSO_4_, 10 mm MgCl_2_, and 20 mm glucose) at 37°C for 60 min. Cells were then diluted in 500 mL LB supplemented with tetracycline (15 *μ*g/mL). Library size was determined by spreading aliquots from each transformation mixture onto LB agar plates (LB containing 15 g agar/liter) supplemented with tetracycline. Libraries were then amplified by incubation overnight (O/N) at 37°C.

Effective library sizes varied between 2.4 × 10^5^ and 3.1 × 10^6^ transformants with an average of 1.1 × 10^6^ transformants. Ten percent glycerol stocks of the amplified libraries were stored at −80°C. Given the observed mutational load of 1.8 errors per sequence (based on sequencing 48 nonselected transformants) and the average library size, one expects that the transformation mixture contains at least one copy of all 2583 one-step mutants, 28% of all possible double mutants, and only 0.04% of all possible triple mutants (http://guinevere.otago.ac.nz/cgi-bin/aef/pedel.pl).

### Selection of mutant alleles

Libraries were exposed to the antibiotics in a selection series of glass bottles containing 50 mL Mueller-Hinton medium (Merck, Whitehouse Station, NJ, USA). Depending on the selection regime, bottles were supplemented with CTX (Sigma-Aldrich, St. Louis, MO, USA; stock solution in 0.1 M NaPO_4_, pH 7.0) or CAZ (Sigma-Aldrich; stock solution in 0.1 M NaPO_4_, pH 7.0) at twofold increments. Concentrations ranged from 0.0625 to 256 *μ*g/mL for CTX and from 1 to 2048 *μ*g/mL for CAZ. Expression was induced by adding 50 *μ*m isopropyl-*β*-D-thiogalactopyranoside (IPTG), thereby mimicking natural expression levels (Barlow and Hall 2002). Expression of cloned TEM alleles in pACSE3 is under control of the *pTac* promoter that is regulated by the lac repressor, which is encoded by the *lacI* gene on the plasmid. Bottles were inoculated with the equivalent of 10× the library size. This ensures that all mutants in the library are present at least once in each bottle. Bottles were incubated for 48 h (37°C). The bottle with the highest concentration of antibiotics in which growth was visible was picked and the bacterial culture was plated on LB-tetracycline agar (O/N; 37°C). We picked a single colony from this plate and grew it in LB-tetracycline (O/N; 37°C). Plasmids were harvested using the GeneElute Plasmid Miniprep Kit (Sigma). These plasmids were then used as template for the next round of mutagenesis and selection.

### Sequencing of TEM

The TEM locus was sequenced with the P3 primer using the BigDye sequencing kit (PerkinElmer, Waltham, MA, USA). Sequences were analyzed using MEGA 5.05 software. Identified nonsynonymous mutations were numbered according to Ambler et al. (Ambler et al. 1991).

### Resistance measurements

For each mutant, we determined the minimum inhibitory concentration (MIC) of CTX and CAZ in triplicate. To exclude spontaneous chromosomal *E. coli* mutations that may have occurred during the selection procedure, plasmids were retransformed into a new batch of DH5*α*E cells prior to the MIC assay. Cultures were then diluted at a titer of 10^5^ cells/mL into in Mueller-Hinton II medium containing 50 *μ*m IPTG in a series of microtitre plates with a twofold increase in CTX or CAZ concentrations ranging from 0.00625 to 4096 *μ*g/mL. Cultures were incubated at 37°C for 24 h. The MIC is the lowest antibiotic concentration without visible bacterial growth.

### Construction of specific mutants

Specific mutations were introduced to TEM-1 by running successive rounds of site-directed mutagenesis using the QuikChange Site-Directed Mutagenesis Kit (Stratagene). We verified whether the desired mutations were incorporated by sequencing the TEM locus. Plasmids were then introduced into *E. coli* strain DH5αE by transformation.

### Statistical analyses

As MIC values are measured on a discontinuous scale (using twofold increases in antibiotic concentration), nonparametric tests on differences in median MIC estimates of replicate lines per treatment were used. Hamming distances among the six final alleles (i.e., the number of amino acid mutations in which they differ) within versus between treatments are tested with one-tailed two-sample *t*-tests based on all possible pairwise distances. The level of specialization toward CTX or CAZ resistance of alleles is calculated as ((increase in CTX resistance)/(0.5 × (increase in CTX resistance + increase in CAZ resistance)) − 1)× 100%; 95% confidence intervals given in Fig.4 have been calculated based on the standard error for the six replicate lines per treatment.

## Results

### Expectation and experimental design

*Escherichia coli* cells that harbor plasmid-borne TEM-1 have promiscuous activity toward third-generation cephalosporins CTX and CAZ: the minimal inhibitory concentrations (MIC) for these antibiotics are 0.125 and 1 *μ*g/mL, respectively. The chemical structures of CTX and CAZ differ from that of ampicillin, for which TEM-1 has high activity, and are larger molecules due to the presence of bulky side groups, which are particularly large in CAZ (Fig.1A). Due to these structural differences, and because CTX and CAZ were found to select preferentially for different mutations in TEM-1, we anticipate negative sign or magnitude pleiotropic effects of mutations on resistance to both antibiotics. In particular, mutation G238S was found to dominate initial adaptation to CTX (Salverda et al. 2011; Schenk et al. 2013), while mutation R164S occurred during adaptation to CAZ (Blazquez et al. 2000). We tested the effects of these mutations on CTX and CAZ resistance and confirmed the asymmetry of their resistance effects toward both antibiotics. Both mutations show magnitude pleiotropy in the background of TEM-1, with G238S having a larger effect on CTX resistance and R164S a larger effect on CAZ resistance, whereas they show sign pleiotropy in the presence of certain other mutations (Table 1). At the same time, these mutations show negative epistasis in their effect on single antibiotic resistance, resulting in sign epistasis for CAZ and reciprocal sign epistasis for CTX resistance (Poelwijk et al. 2007) (Table 1).

**Table 1:** TEM-1 mutants and the minimum inhibitory concentrations (MIC) of CTZ and CAZ. Mutants were constructed using site-directed mutagenesis. Bold underlined values represent the peak(s) in each two-locus fitness landscape; landscapes with reciprocal sign epistasis have two peaks. Mutations E104K, M182T, and T265M do not influence the occurrence of sign epistasis on CAZ, but do change the occurrence of reciprocal sign epistasis on CTX

Mutant	MIC CTX (*μ*g/mL)	MIC CAZ (*μ*g/mL)
TEM-1	0.125	1
G238S	**4**	2
G238S + R164S	0.25	8
R164S	**1**	**32**
TEM-1	0.125	1
G238S + E104K	**64**	64
G238S + R164S + E104K	8	256
R164S + E104K	**16**	**1024**
TEM-1	0.125	1
G238S + M182T	**32**	4
G238S + R164S + M182T	4	32
R164S + M182T	1	**64**
TEM-1	0.125	1
G238S + E104K + M182T	**512**	256
G238S + R164S + E104K + M182T	32	512
R164S + E104K + M182T	16	**4096**
TEM-1	0.125	1
G238S + T265M	**16**	4
G238S + R164S + T265M	1	8
R164S + T265M	**2**	**32**
TEM-1	0.125	1
G238S + E104K + T265M	**256**	64
G238S + R164S + E104K + T265M	32	256
R164S + E104K + T265M	16	**2048**

**Figure 2:**
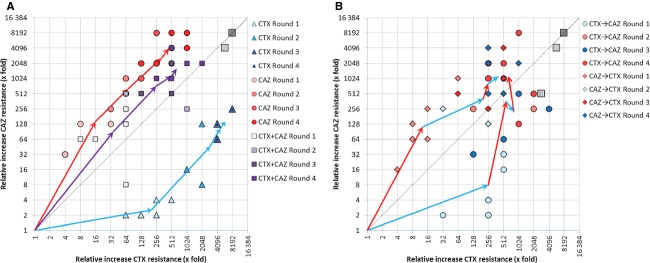
Improvement in MIC of TEM-1 for CTX and CAZ during four rounds of evolution for all five experimental conditions. The arrows display the mean trajectory for each regime, and symbols show the individual values for each replicate line (blue for CTX, red for CAZ, and purple for the combined CTX + CAZ selection, from light to dark for round 1 to 4). (A) Adaptation in response to selection with CAZ and CTX alone, and in response to a combined exposure. (B) Adaptation in response to selection regimes in which CTX and CAZ are alternated, starting with either CAZ or CTX. The big gray squares indicate the maximum (dark) and median (light) expected resistance after four rounds of selection in the absence of pleiotropy; the shaded square in panel B gives this expectation for the fluctuating selection lines after comparable selection with each antibiotic (i.e., two rounds). Individual trajectories of experimental lines are given in Fig. S1A–E.

To test for the role of pleiotropy during adaptation of TEM-1 to CTX and CAZ, we used *in vitro* evolution of TEM-1 under five experimental conditions: in the presence of CTX alone, CAZ alone, CTX and CAZ combined, and CTX and CAZ alternated with either CTX or CAZ as the first antibiotic (Fig.1B). For each condition, six independent replicate lines were subjected to four rounds of error-prone PCR followed by the selection in a range of concentrations of one or both antibiotics. After each round, a single clone was isolated from the bottle with the highest antibiotic(s) concentration that still permitted bacterial growth. Plasmids from this clone were isolated, sequenced, and subjected to a new round of error-prone PCR and selection. Figure2 shows the CTX and CAZ MIC estimates of all 30 lines during the four rounds of evolution (after transformation into an isogenic background), and Table 2 and Table S1 show the selected mutations.

**Table 2:** Parallel amino acid substitutions in TEM-1 *β*-lactamase alleles evolved toward increased CTX or CAZ resistance. Substitutions are numbered according to codon number in TEM-1 (Ambler et al. 1991) with single-letter codes for the ancestral (left) and the new amino acid. The colors indicate whether mutations were selected with CTX (blue), CAZ (red), or the combination CTX + CAZ (purple), and darkness indicates in which round they were selected (light in first to dark in fourth round). Mutations that were unique to a single line and silent mutations are listed in Table S1. The total number of selected mutations (including unique and synonymous mutations)and the MIC of the final alleles are listed at the bottom of table

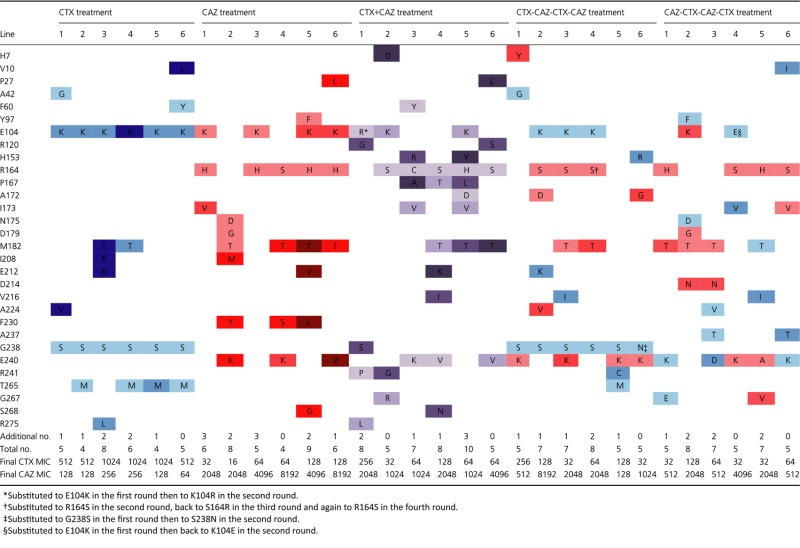

### Selection in the presence of CTX or CAZ

**Figure 3:**
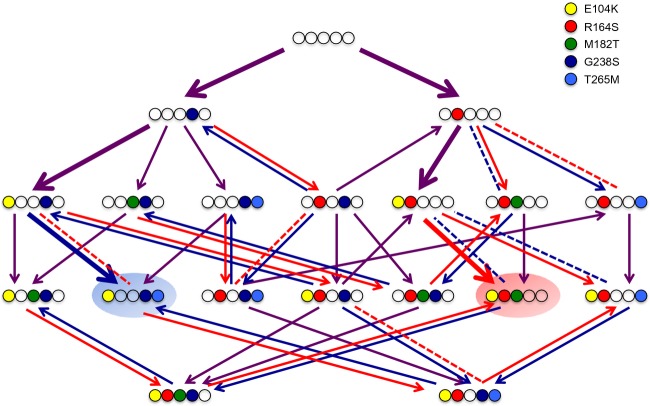
Arrow plot for five frequently observed mutations based on the MIC values for CTX and CAZ of the mutants presented in Table 1. The five mutations are represented by differently colored circles, as indicated in the legend. Arrows point toward higher MIC values for CTX (blue) and CAZ (red) or both (purple); dashed lines connect genotypes with equal MIC. Bold arrows indicate the two dominant pathways of three mutations selected by CTX and CAZ mono-therapy (involving G238S-E104K-T265M highlighted in blue for CTX and R164S-E104K-M182T highlighted in red for CAZ, see Table 2). Switching between CTX and CAZ selection does not cause the cycling between more than two genotypes, leading to sustained antibiotic susceptibility for one antibiotic.

We first ask whether mutations that are selected in the presence of CTX or CAZ show pleiotropic effects with respect to the other antibiotic. The selected alleles display large increases in resistance for both antibiotics in the first round, followed by smaller increases in later rounds (Fig.2A). The adaptive potential of TEM-1 toward both antibiotics is similar, as the final increases in MIC are not significantly different (median increase in MIC for CTX = 5792-fold, for CAZ = 4032-fold; Mann–Whitney *U* = 12, *P* > 0.10). Yet, the CTX- and CAZ-selected alleles use largely different sets of mutations (Table 2 and Table S1). The CTX lines share several mutations: All lines carry G238S and E104K, often in combination with T265M (see bold arrows in Fig.3). The six CAZ lines have accumulated similar numbers of nonsynonymous mutations as the CTX lines (CTX: 5.2, CAZ: 6.3 substitutions; *U* = 10, *P* > 0.10), although they partly differ in the type of mutations: five of the six lines carry R164S or R164H and a combination of mutations occurring at amino acid positions 104, 182, or 240 (see bold arrows in Fig.3). One line deviates from this pattern (line 2, see Table 2) and harbors a mutation at position 179 instead of position 164 in the omega loop. As a formal test of the difference in the type of mutations selected by CTX and CAZ, we calculated the Hamming distance (as the sum of amino acid-site differences) of the six round-4 alleles within and between both selection regimes. Hamming distances are significantly larger between (6.8) than within selection regimes (3.4 for CTX, *t* = 5.44, d.f. = 49, *P* < 0.0001; 5.3 for CAZ, *t* = 2.34, d.f. = 49, *P* = 0.012), supporting the selection of different mutations by these two antibiotics.

**Figure 4:**
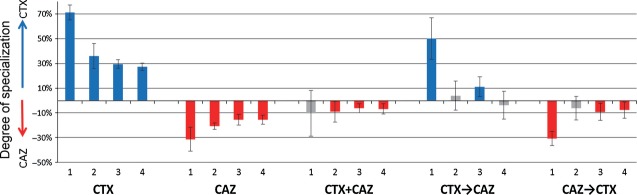
Degree of specialization of the selected lines on either CTX (positive values) or CAZ (negative values) during four rounds of selections. See methods for how specialization has been calculated. Error bars represent 95% confidence intervals. Blue bars indicate specialization on CTX, red bars indicate specialization on CAZ, and gray bars indicate the absence of significant specialization.

A comparison of direct (i.e., resistance improvement for the antibiotic used for selection) and correlated responses (i.e., resistance improvement for the other antibiotic) shows that the selected mutations have weak pleiotropic effects, because correlated responses are positive in both cases (Fig.2A). However, the direct response always exceeds the correlated response (respectively, 5792- vs 128-fold increase in CTX-selected lines, *U* = 0, *P* < 0.01; 4032- vs 512-fold increase in the CAZ-selected lines, *U* = 0, *P* < 0.01). Moreover, as was the case for single mutations G238S and R164S (Table 1), the correlated response after four rounds of evolution is asymmetric: Selection for CAZ resistance is accompanied by larger increases in CTX resistance than vice versa for selection with CTX, as shown by their level of specialization toward CTX or CAZ (Fig.4). Interestingly, specialization largely halts after the first round, and mutations selected in later rounds are approximately equally beneficial for CTX and CAZ resistance (Figs2A and 4). This pattern is supported by the observed sequence of mutations (Table 2), where initial mutations G238S (CTX) and R164S (CAZ) are involved in increasing activity toward the new substrate, while they decrease thermodynamic stability of the enzyme (Wang et al. 2002). These mutations are followed by mutations such as M182T, E104K, and T265M, which have smaller or no effect in the original background, but are global suppressors of TEM-1 instability that increase resistance in the background of the initial mutations (Wang et al. 2002; Salverda et al. 2010).

### Selection in the simultaneous presence of CTX and CAZ

We next ask to what extent pleiotropy constrains adaptation of TEM-1 when exposed to both antibiotics simultaneously. To this end, we evolved six lines during four rounds of mutagenesis and selection in a gradient of CTX and CAZ, present at a constant ratio of 1:8, which reflects the difference in MIC of TEM-1. As can be seen in Fig.2A, the median increase in MIC of the combined-antibiotic treatment is significantly lower than the direct response of the single antibiotic lines (512- vs 5792-fold increase for CTX, *U* = 0, *P* < 0.01; 1448- vs 4032-fold increase for CAZ, *U* = 5, *P* < 0.05). Thus, constraints from pleiotropy on adaptation also occur under stringent selection in the presence of both antibiotics, but they are not so strong as to prevent the evolution of resistance to either antibiotic.

In addition, the MIC trajectories of the CTX+CAZ lines show an asymmetric response that reflects the influence of pleiotropy (Fig.2A). After the first two rounds, MIC values resemble those of the CAZ lines for both CTX (*U* = 10.5, *P* > 0.10) and CAZ resistance (*U* = 15.5, *P* > 0.10), but are different from those of the CTX lines (lower for CTX: *U* = 2, *P* < 0.01; higher for CAZ: *U* = 0, *P* < 0.01). Nevertheless, the degree of specialization is close to zero (Fig.4), and only slightly biased toward CAZ specialization. The pattern of substitutions supports the resemblance with the CAZ monotherapy lines: The majority of the lines use mutations at the same positions as the CAZ-selected lines, including at key position 164 (Table 2), but mutations are also observed at new positions, such as 120, 153, 167, and 241. This greater resemblance of the coselected lines to the CAZ lines is supported by the lower Hamming distance of their final alleles to those of these lines (6.8) relative to the final alleles to the CTX lines (7.9, *t* = 1.87, d.f. = 70, *P* = 0.033). One line deviates from this pattern and follows a trajectory that more closely resembles the CTX lines, involving G238S. The total number of mutations selected under CTX+CAZ conditions is not different from that under single antibiotic selection (7.0 vs 5.2 for CTX, *U* = 7.5, *P* > 0.10, and 6.3 for CAZ, *U* = 14.5, *P* > 0.10).

### Selection in the fluctuating presence of CTX and CAZ

Finally, we investigate the effect of fluctuating selection on resistance evolution. Six lines were subjected to alternating selection starting with CTX, and six other lines started with CAZ. MIC trajectories resemble the trajectories of the single antibiotic lines for the antibiotic used during the first round, but later converge to similar MIC values for both antibiotics (Fig.2B). The level of specialization that occurs during the first round disappears in later rounds and resembles that of the CTX+CAZ lines (Fig.4).

As can be seen from the mean (Fig.2B) and the individual trajectories (Fig. S1) of the lines starting with CTX selection, here some of the selected mutations show sign pleiotropy. To test for the influence of pleiotropic constraints (i.e., any deviation from a perfect correlated response), we compare the final resistance of the fluctuating selection lines with the direct response of the single antibiotic lines. We find clear pleiotropic constraints on CTX improvement (724- and 362- vs 5792-fold increase in MIC, *U* = 0 and *U* = 0, both *P* < 0.01) and weaker constraints on CAZ improvement (1448- and 1024- vs 4032-fold increase in MIC, *U* = 7, *P* < 0.10 and *U* = 5, *P* < 0.05). Improvements in resistance of the fluctuating selection lines are even lower than expected without pleiotropy (i.e., relative to the single antibiotic lines after two rounds of selection) for CTX (645- and 406- vs 2580-fold increase in MIC, *U* = 3 and *U* = 0.5, *P* < 0.01 for both), but not for CAZ (1149- and 1149- vs 645-fold increase in MIC, *U* = 12, *P* > 0.10), and similar to those of the CTX+CAZ lines (724-/362- vs 512-fold increase in CTX resistance, *U* = 18 and *U* = 11, both *P* > 0.10; 1448-/1024- vs 1448-fold increase in CAZ resistance, *U* = 15.5 and U = 13.5, both *P* > 0.10).

Consistent with the pattern seen in the MIC trajectories (Fig.2), the pattern of substitutions also indicates the contingency of adaptation on the antibiotic used in the first round of evolution. Not only do they use similar mutations as the single antibiotic lines in round 1 (Table 2), but these first-round mutations also partly determine the mutations used in later rounds. This pattern of resemblance is supported by the lower Hamming distances of final alleles from the fluctuation lines with the monotherapy lines of the antibiotic used in the first round than those starting with the other antibiotic: 5.2 vs 6.5 for the lines starting with CTX (*t* = 2.72, d.f. = 70, *P* = 0.0041) and 5.8 vs 7.8 for the lines starting with CAZ (*t* = 4.66, d.f. = 70, *P* < 0.0001).

A remarkable observation is the co-occurrence of negatively interacting mutations G238S and R164S in three fluctuating selection lines starting with CTX: In lines 2 and 3, they remain together for three rounds, while in line 4, mutation R164S reverses in the third round, but is selected back in the fourth round (Table 2). Table 1 shows that the addition of R164S to alleles containing G238S is beneficial in the presence of CAZ, but the addition of G238S to alleles containing R164S is not beneficial in the presence of CTX, consistent with their sequential selection only in fluctuating selection lines started with CTX. However, their co-occurrence during three rounds in two lines raises the possibility that other mutations may have alleviated the negative interaction between these mutations and prevented their reversion. Mutations shared by lines selected with CTX and CAZ separately are candidates for such compensatory effects. To test this possibility, we constructed mutants containing G238S and R164S together with E104K, M182T and T265M. MIC measurements confirm that M182T alone and the combination T265M and E104K ameliorate the negative interaction between G238S and R164S and change it from reciprocal sign epistasis to sign epistasis in the presence of CTX (Table 1). Nevertheless, reversion S164R remains beneficial in all tested genetic backgrounds when switching from CAZ back to CTX in round 3, suggesting additional ameliorating effects from other mutations selected in these lines.

## Discussion

The diverse effects of single mutations on multiple phenotypic traits, called pleiotropy, are a key feature of the genotype–phenotype map with fundamental evolutionary consequences (Fisher 1930; Otto 2004; Østman et al. 2011; de Visser et al. 2011; Wagner and Zhang 2011). Previous work has revealed pervasive pleiotropy affecting traits at all levels of biological organization, from complex traits and diseases in humans (Solovieff et al. 2013) to properties of single enzymes (DePristo et al. 2005; Soskine and Tawfik 2010). However, tests of pleiotropy are often weak and based on correlated effects on traits that are not related to fitness under the selective conditions. Finding trade-offs in these tests does not rule out that mutations without or with smaller negative pleiotropic effects exist, and hence, pleiotropic constraints under selection for both traits may still be limited. In addition, they often lack information about the genetic changes involved, allowing alternative explanations than sign pleiotropy for observed negative correlations, for example, the accumulation of mutations with neutral effect under the selective conditions and deleterious effect on the correlated trait (Medawar 1952; Williams 1957).

We tested for the influence of pleiotropic constraints during the evolution of two novel functions of the enzyme TEM-1 *β*-lactamase, that is, the activity on the *β*-lactam antibiotics CTX and CAZ. We expected the influence of negative pleiotropy based on the different molecular structures of these antibiotics and the previously observed selection of different mutations by these antibiotics (Blazquez et al. 2000; Salverda et al. 2011). TEM-1 rapidly adapts to these novel antibiotics, as selection with a single antibiotic results in enzymes with an up to 8000-fold increased resistance level caused by a handful of mutations after just four rounds of *in vitro* evolution. Our results show that pleiotropic constraints are relatively weak and that TEM-1 also evolves high resistance levels when both antibiotics are used simultaneously or alternatingly. The limited negative pleiotropy that we observed is perhaps not surprising, given that TEM-1's activity on CTX and CAZ is already very low and possibilities to detect sign pleiotropy, involving further decreases in activity on either antibiotic, are limited. In addition, CTX and CAZ share the same basic structure of a *β*-lactam ring with bulky side groups. Selection with *β*-lactam antibiotics with larger structural differences such as ampicillin and cefotaxime (Fig.1A), or with a combination of an antibiotic and a *β*-lactamase inhibitor such as clavunalate (Vakulenko and Golemi 2002; Salverda et al. 2010), may result in stronger pleiotropic constraints.

Nevertheless, pleiotropic constraints were observed. We found evidence for sign pleiotropy for individual mutations that were frequently observed in the selected alleles (Table 1 and Fig.3): Nine of the 34 (∼26%) resistance effects of single mutations that were measured showed sign pleiotropy. In addition, a comparison of the final alleles from the different selection regimes revealed mostly magnitude pleiotropy. First, selection with a single antibiotic resulted in specialization, that is, the direct response (resistance improvement on the antibiotic used for selection) always exceeded the correlated response (resistance improvement on the other antibiotic). Second, after four rounds of selection, when adaptation has slowed down considerably, alleles selected in the simultaneous presence of both antibiotics reached resistance levels which are significantly below those of the single antibiotic lines. In other words, selection with two antibiotics results in generalist enzymes which are good at performing both novel functions, but not as good as a specialized enzyme would be, supporting the notion that ‘the jack of all trades is the master of none’ (MacArthur 1972).

Third, we tested whether adaptation in a fluctuating environment might overcome some of these constraints. A fluctuating environment may reveal pleiotropic constraints from the fluctuating selection of alternative adaptive phenotypes in each environment, but may in contrast also allow a temporal release from epistatic constraints experienced in the presence of a single antibiotic where an allele may be ‘stuck’ on a local fitness peak (Weinreich et al. 2006; Salverda et al. 2011; Schenk et al. 2013). Such a release from epistastic constraints has been observed for HIV adapting to two different T-cell lines, where adaptation in one cell environment led to a higher fitness in the alternative than in the selective cell environment (van Opijnen et al. 2007). However, we found no support for this scenario. Rather, resistance levels were comparable to those of alleles selected in the simultaneous presence of both antibiotics and substantially lower than those selected with single antibiotics.

We speculate that the relatively subtle pleiotropy observed in our study results from structural changes involved in accommodating and positioning CTX and CAZ with respect to catalytically important residues, such as Ser-70 and Glu-166 (Salverda et al. 2010). The presence of the different side groups influences the positioning of the antibiotic molecule relative to these catalytic residues, and maximal catalytic efficiency requires a slightly different conformation for each antibiotic. Being exposed to two antibiotics with different side groups likely selects for a compromise conformation that is suboptimal for each antibiotic individually. We have two reasons to believe that changes in the size of the oxyanion pocket surrounding the active site (Ser-70) mediate the observed pleiotropy. First, both CTX and CAZ are larger than penicillin, and increasing the size of the cavity is beneficial for resistance to both antibiotics, which may explain the correlated response that we observed. Second, selection for CAZ resistance alone is accompanied by a stronger correlated response in CTX resistance than vice versa. This asymmetry is consistent with the notion that selection with CAZ results in a larger cavity that easily fits the smaller CTX molecule, whereas selection with CTX results in a cavity that is still small for the CAZ molecule. Direct structural analysis of TEM-1 mutants G238S and R164S may resolve the possible role of the size of the oxyanion pocket.

However, we cannot rule out that uncontrolled differences in selective conditions between the different antibiotic regimes contribute to the observed pattern of pleiotropy. For instance, the lower improvement of the combined selection lines relative to the single antibiotic lines may be partly caused by lower antibiotic concentrations in cultures from which bacteria were selected, because the concentration of each may be lower due to their combined bactericidal effect. Differences in selective conditions may also contribute to the weak asymmetry in the response of the combined selection lines. While we kept the ratio of CTX/CAZ concentration at 1:8, reflecting the difference in TEM-1's MICs, this balance in selection pressures may have changed after the substitution of the first mutations. Given the resemblance of the final resistance improvement of the combined selection lines with the fluctuating selection lines (see Fig.2) – where the problem of imbalanced selection pressures was absent, we do not think that uncontrolled differences in selective conditions have been a very important factor.

At least two factors limit the translation of our results to clinical situations. First, the pleiotropic constraints that we observed during adaptation of a single enzyme to two related cephalosporins do not necessarily reflect similar constraints when more genes or different classes of antibiotics are involved. Resistance mutations generally reduce bacterial fitness in the absence of the same drug (Andersson and Hughes 2010), but still little is known about pleiotropic effects in the presence of other antibiotics, especially when multiple resistance mechanisms are involved (but see (Imamovic and Sommer 2013)). Resistance to drugs interacting with different cellular targets is likely associated with weaker pleiotropic constraints than to drugs with a common target. Nonetheless, the indirect effects of different drug–target interactions on common cellular responses, such as SOS response, DNA repair, and cell death pathways, suggest ample substrate for pleiotropy (Kohanski et al. 2010).

Second, the fact that our study is restricted to only two different antibiotics limits its relevance to assessing strategies for containing the evolution of antibiotic resistance through the alternating use (‘cycling’) of different antibiotics (Yeh et al. 2009; MacLean et al. 2010; Goulart et al. 2013; Imamovic and Sommer 2013; Palmer and Kishony 2013). Goulart et al. (2013) addressed this problem by analyzing the resistance landscapes of two TEM-1 *β*-lactamase mutants in the presence of 15 different structurally similar antibiotics, including CTX and CAZ. Using a computational approach, they identified pathways through those landscapes where selection for resistance to three or four alternating antibiotics could lead to repeated cycles among limited sets of alleles, thus sustainably renewing the usefulness of these antibiotics.

With the alternating use of only two antibiotics, any cycle necessarily has to switch between two alleles. Inspection of the resistance landscape in Fig.3 shows that it contains several such simple cycles. For example, the pathway connecting local resistance maxima E104K + G238S + T265M and E104K + R164S + T265M through the gain of R164S and subsequent loss of G238S under selection with CAZ reverts its direction when selecting with CTX. Whereas this pathway is not observed in any of the lines undergoing fluctuating selection, the repeated reversion of R164S in line 4 of the CTX>CAZ>CTX>CAZ regime may be seen as small-scale support for the approach advocated by Goulart et al. It should be noted, however, that the second substitution of R164S in the fourth round of selection is accompanied by the substitution of M182T, which implies that evolution is not truly cyclic. This illustrates the general difficulty of keeping the adapting population within the small set of alleles that constitute the desired cyclic trajectory.

Whether for designing cycling drug therapies or for designing strategies involving the simultaneous application of drug combinations (Yeh et al. 2009; Pena-Miller et al. 2013; Jansen et al. 2014), understanding the pleiotropic and epistatic properties of resistance mutations will be imperative (Schenk and De Visser 2013). We hope that our finding of limited pleiotropic constraints from the combined and fluctuating use of two related antibiotics interacting with a single target, together with strong epistatic constraints in the presence of single antibiotics (Salverda et al. 2011; Schenk et al. 2013), will motivate further research in these directions.

## Data archiving

Data for this study are available at the Dryad Digital Repository: http://doi.org/10.5061/dryad.q8635

## Supporting Information

**Figure S1.** Improvement in MIC for both antibiotics during four rounds of mutagenesis and selection for each replicate line in response to selection with (A) CTX alone, (B) CAZ-CTX alone, (C) CTX and CAZ combined, and regimes in which CTX and CAZ are alternated, starting with either (D) CTX or (E) CAZ.

**Table S1.** Amino acid substitutions and silent mutations in TEM β-lactamase alleles evolved towards increased CTX or CAZ resistance.
