# Supplementary figures and images for: Role of pleiotropy during adaptation of TEM-1 β-lactamase to two novel antibiotics
Source: Evol Appl. 2014 Sep 18;8(3):248–60. doi: 10.1111/eva.12200 (PMC4380919; doi:10.1111/eva.12200)

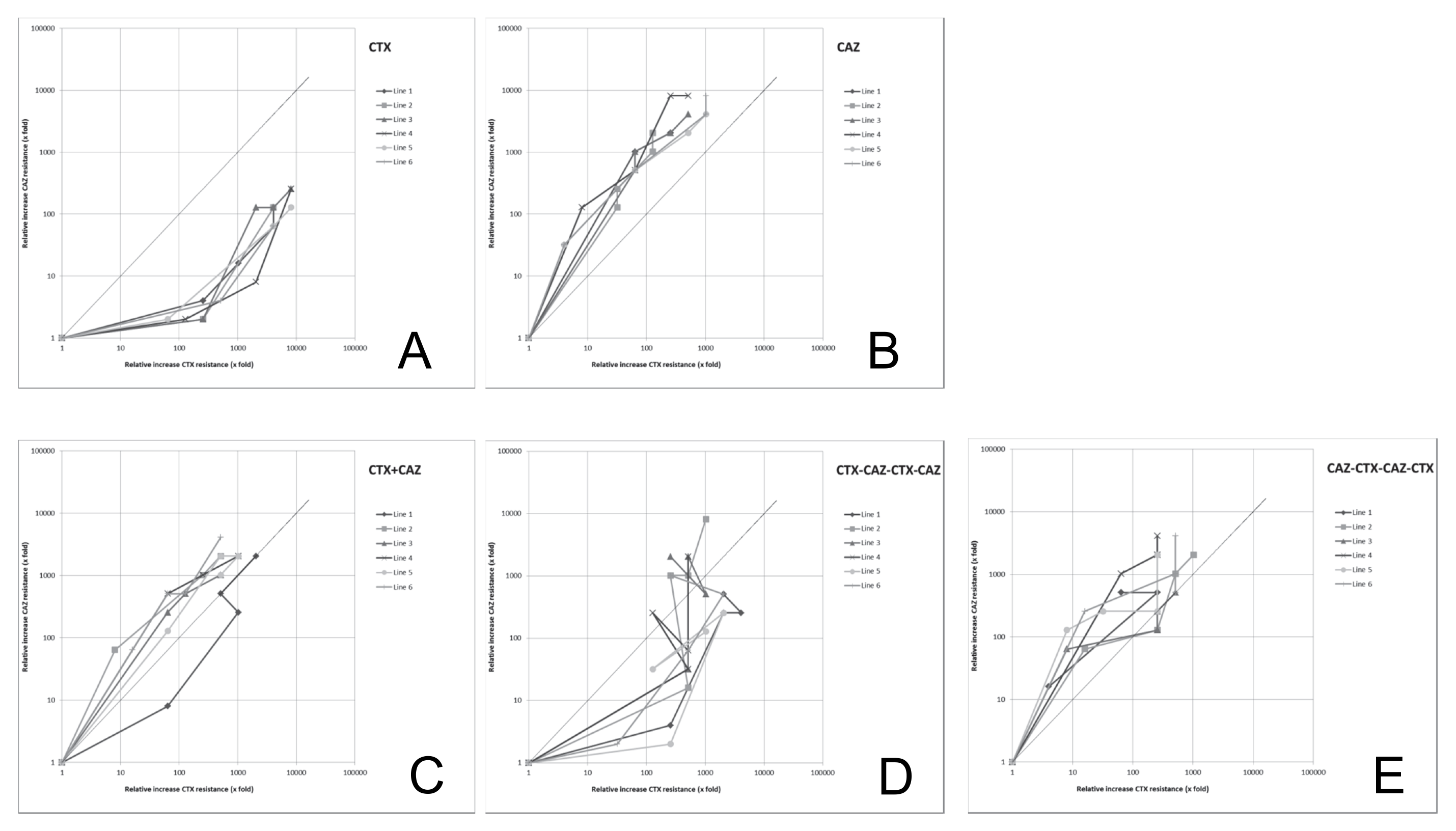

Supplement: Supplementary file 1 — Figure S1. Improvement in MIC for both antibiotics during four rounds of mutagenesis and selection for each replicate line in response to selection with (A) CTX alone, (B) CAZ-CTX alone, (C) CTX and CAZ combined, and regimes in which CTX and CAZ are alternated, starting with either (D) CTX or (E) CAZ. [file eva0008-0248-sd1.tif]
