# Supplementary material for: Role of pleiotropy during adaptation of TEM-1 β-lactamase to two novel antibiotics
Source: Evol Appl. 2014 Sep 18;8(3):248–60. doi: 10.1111/eva.12200 (PMC4380919; doi:10.1111/eva.12200)
Supplement: Supplementary file 2 — Table S1. Amino acid substitutions and silent mutations in TEM β-lactamase alleles evolved towards increased CTX or CAZ resistance. [file eva0008-0248-sd2.docx]

**Supplementary material**

**Table S1**

Amino acid substitutions and silent mutations in TEM β-lactamase alleles evolved towards increased CTX or CAZ resistance. Amino acid substitutions are numbered according to codon number in TEM-1 ([Ambler et al. 1991](#_ENREF_1)) with single-letter codes for ancestral (left) and new (right) amino acids. Silent mutations are in italics and numbered according to the nucleotide positions in TEM-1 with single-letter codes for the ancestral (left) and the new (right) nucleotide.

| Line | 1 | 2 | 3 | 4 | 5 | 6 |
| --- | --- | --- | --- | --- | --- | --- |
| CTX treatment | Q6H | L49M | L198V | R277V | P62A | *g81a* |
|  | t108c | *a75t* | A227V | V277W |  |  |
|  | *t675g* | *a222t* | *c72t* | *t539c* |  |  |
|  |  | *g84a* | *c139t* |  |  |  |
| CAZ treatment | A18T | T189M | I13F | *c18t* | A36S | F8L |
|  | R191H | I282M | Q39R |  | L51I | *t600a* |
|  | A270S | *g306a* | Q269H |  | *g219c* |  |
|  | *a511g* | *c441a* |  |  | *g717a* |  |
|  | *g678a* | *g336a* |  |  |  |  |
| CTX+CAZ treatment | F24Y | *a738t* | T140I | E110K | D35N | *a183g* |
|  | E197K | *c762a* | *a348g* | *g114a* | E58G | *g336a* |
|  |  |  | *g168a* | *c378t* | E272V |  |
|  |  |  |  | *t690g* | *t15c* |  |
|  |  |  |  | *c297t* |  |  |
| CTX-CAZ-CTX-CAZ treatment | M129L | A249V | R61H | I47V | A184V | t15c |
|  | *t51c* | *a351t* | *g231a* | L201V | *c27t* | *c27t* |
|  | *c297t* | *c240g* | *a471g* | *c453t* |  |  |
|  | *t801c* |  | *t465c* | *a813t* |  |  |
|  | *c477t* |  |  | *a546c* |  |  |
|  |  |  |  | *a471g* |  |  |
| CAZ-CTX-CAZ-CTX treatment | W165R | R178L | L113H | *c768t* | G41D | *g429a* |
|  | *c204t* | A217T | D163G | *t30c* | M155L |  |
|  | *g411a* | *g663t* |  |  | *a195t* |  |
|  |  |  |  |  | *c660a* |  |
